# Analysis of Environmental and Social Significant Factors Affecting the Flow of Maternal Patients in Jilin, China

**DOI:** 10.3389/fpubh.2022.780452

**Published:** 2022-05-09

**Authors:** Dongmei Mu, Hua Li, Dongxuan Wang, Xinyu Yang, Shutong Wang

**Affiliations:** ^1^Department of Clinical Research, The First Hospital of Jilin University, Changchun, China; ^2^Department of Abdominal Ultrasound, The First Hospital of Jilin University, Changchun, China; ^3^School of Public Health, Jilin University, Changchun, China

**Keywords:** maternal patients, environmental factors, social factors, significant factors, temperature, entrance, busy farming, festivals

## Abstract

**Background:**

With the implementation of China's Two-child policy, the number of pregnant women has been increasing year by year in recent years. However, the pregnancy success rate of pregnant women is declining year by year, and it is almost necessary for all the elderly mothers to do pregnancy protection.

**Objective:**

The purpose of this study is to analyze the social and environmental factors that affect the patient flow of pregnant women in Jilin area of China, and further utilize the favorable factors to avoid the negative effects of adverse factors, so as to improve the pregnancy success rate and eugenics level.

**Methods:**

Monthly patient flow data from 2018 to 2020 were collected in the obstetrics department of the First Hospital of Jilin University. The decompose function in R software was used to decompose the time series data, and the seasonal and trend change rules of the data were obtained; the significant factors influencing patient flow were analyzed by using Poisson regression model, and the prediction model was verified by using assumptions, such as the normal distribution of residuals and the constant difference of residuals.

**Results:**

Temperature in environmental factors (*P* = 4.00E−08) had a significant impact on the flow of obstetric patient. The flow of patients was also significantly affected by the busy farming (*P* = 0.0013), entrance (*P* = 3.51E−10) and festivals (*P* = 0.00299). The patient flow was accompanied by random flow, but also showed trend change and seasonal change. The trend of change has been increasing year by year. The seasonal variation rule is that the flow of patients presents a trough in February every year, and reaches the peak in July.

**Conclusion:**

In this article, Poisson regression model is used to obtain the social and environmental significant factors of obstetric patient flow. According to the significant factors, we should give full play to significant factors to further improve the level of eugenics. By using time series decomposition model, we can obtain the rising trend and seasonal trend of patient flow, and then provide the management with decision support, which is conducive to providing pregnant women with higher level of medical services and more comfortable medical experience.

## Introduction

Previous studies have shown that infants, children and pregnant women are susceptible to climate change and air pollution (1, 2). People of childbearing age (especially pregnant women) carry an important initial stage of “early 1,000 days of life.” From the differentiation of germ cells, the combination and implantation of fertilized eggs, to the development and delivery of embryos, the exposure to environmental risk factors of extreme climate change and air pollution at different stages of life gestation will have adverse effects on maternal and infant health (3). Therefore, studying the impact of climate change and air pollution on the risk of various adverse pregnancy outcomes in expectant families and pregnant women has important public health value and social significance.

The issue of climate warming has always been the focus of global attention. Climate warming is not only a problem that the government needs to pay attention to, but also closely related to people's daily life. Wu et al. (4) studied the change of thermal comfort in China. In the past few decades, China's effective temperature continued to rise, and the average number of cold and very cold days decreased by 3.5 days per 10 years, while the number of hot and very hot days increased by 0.7 days per 10 years. A large number of studies worldwide have described the non-linear and delayed relationship between prevalence and ambient temperature. Xu et al. (5) analyzed the effects of extreme body temperatures admitted to pediatric departments in Australia and found that children are particularly vulnerable to the effects of high and low temperatures, which can be seen in a range of pediatric diseases. Hotz et al. (6) used Poisson generalized linear model to study the relationship between daily patient flow and daily average temperature in emergency departments. In order to quantify the relationship between final patient flow and temperature (7), an unconstrained distributed lag model was used to evaluate the relationship. Each lag was modeled separately, and then the effect of all temperatures on patient flow in emergency departments was counted. The threshold temperature is determined by looking at the patient flow chart for each specific cause in turn. A recent systematic review (8) analyzed the effects of high temperature environments on accidental injuries at all ages, and the association between high temperatures and increased hospitalizations for respiratory diseases, kidney diseases, and injuries, with 11 of the 13 disease studies surveyed being associated with high temperatures. The authors further found that this was associated with increased daily temperature, increased sunshine duration, and decreased rainfall.

The association between air pollution and adverse pregnancy outcomes has been widely recognized, including small for gestational age, stillbirth, infertility and birth defects (9). Due to the different social and natural environments in different parts of Africa, the study of environmental factors related to placental diseases will play an important role in the development of effective interventions. When the environmental concentration exceeds the critical value, heavy metals are considered to be harmful factors. Pregnant women can be exposed to a variety of chemicals at home and at work (10), some of which contain endocrine disrupting chemicals. The results showed that mother's occupational exposure to chemicals was a risk factor for hypospadias, and it suggested that the use of hairdressing cosmetics in early pregnancy might affect the incidence of hypospadias in newborns. Cardiovascular disease is the leading cause of patient death worldwide, a large number of studies have shown that environmental factors (11) such as air pollution and temperature changes lead to an increased risk of cardiovascular disease incidence. Tian et al. (12) have used the Poisson regression model to link the increase of temperature with the increase of the number of hospitalized patients with cardiovascular diseases and their subtypes. Liu et al. (13) used conditional logistic regression model to study cardiovascular disease in 26 Chinese cities .The results showed that elevated concentrations of sulfur dioxide, nitrogen dioxide, carbon monoxide and ozone were associated with an increased risk of hospitalization for heart failure.

Maternal smoking and secondhand smoke exposure during pregnancy have many negative health effects on both mother and child, which is related to the decline of health level (14, 15). In order to determine the social determinants of maternal smoking and exposure to second-hand smoke during pregnancy, Do et al. (16) used multiple logistic regression to analyze the data of tobacco and healthy population. The results showed that the people with higher risk of mother smoking included those with general/poor mental health status, and those with higher risk of second-hand smoke exposure included younger and earlier pregnant people. Frazer et al. (17) found that maternal smoking is a key modifiable risk factor for preventing adverse pregnancy outcomes, such as intrauterine growth restriction, premature delivery and stillbirth. Reynolds et al. (18) studied the annual trend of mothers' smoking reported at the first antenatal examination of women who delivered in maternity hospitals of large universities over the past 5 years. The number of women who reported smoking at the first antenatal examination decreased year by year. Multiple logistic regression analysis was used to determine the maternal characteristics, health behaviors and mental history related to smoking behavior. Ngo et al. (19) conducted a cross-sectional study on 432 pregnant women to assess the prevalence of second-hand smoke exposure among non-smoking pregnant women, collected the socio-economic characteristics and second-hand smoke exposure information of participants, and multivariate logistic regression analysis was used to determine the related factors. The results showed that the proportion of second-hand smoke exposure among non-smoking pregnant women was high, and the main contact places included family, workplace, cafeterias and restaurants.

With the implementation of China's two-child policy in 2016, the number of pregnant women has increased rapidly in recent years. However, the success rate of pregnancy has decreased year by year. The above studies show that there is a significant correlation between environmental factors and the health level of newborns. In addition, with the improvement of people's living standards, the pursuit of high-quality medical resources for pregnant women is also increasing, which puts forward how to better meet the needs of maternal health services. Providing a comfortable environment for pregnant women in terms of environmental factors and providing better medical services for pregnant women have become important ways to improve the success rate of pregnancy and the level of eugenics. In this article, Poisson regression model is used to analyze the social and environmental significant factors that affect the flow of patients, so as to give full play to the significant factors to further improve the success rate of pregnancy and the level of eugenics. The time series decomposition model is used to analyze the trend and seasonal variation of patient flow, which is conductive to providing the management with decision support and providing pregnant women with higher level of medical services and more comfortable medical experience.

## Data and Methods

### Data

The First Hospital of Jilin University, located in Changchun, the capital of Jilin Province, is a representative tertiary hospital with high medical level in Jilin Province. We collected a historical data set of maternal patient flow from January 1, 2018 to February 29, 2020 in the hospital obstetrics department, with a total number of 2,345. Maternal patients refer to patients who go to the hospital to choose natural delivery or cesarean section. We did not use the data before 2018 due to the lack of data. In addition, due to the COVID-19 outbreak, the hospital imposed a patient flow restriction on the obstetrics department, that is, the data in March 2020 were not seasonal and trending, and the data in this period have not been collected. In order to analyze the correlation between patient flow and environmental and social factors in the past 2 years, the data were classified and summarized to get the monthly patient flow, and then a total of 26 data records were obtained.

**Figure 1:**
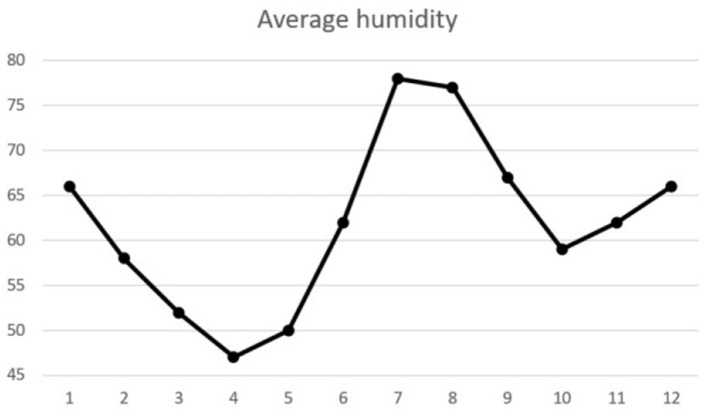
Daily average humidity chart.

**Figure 2:**
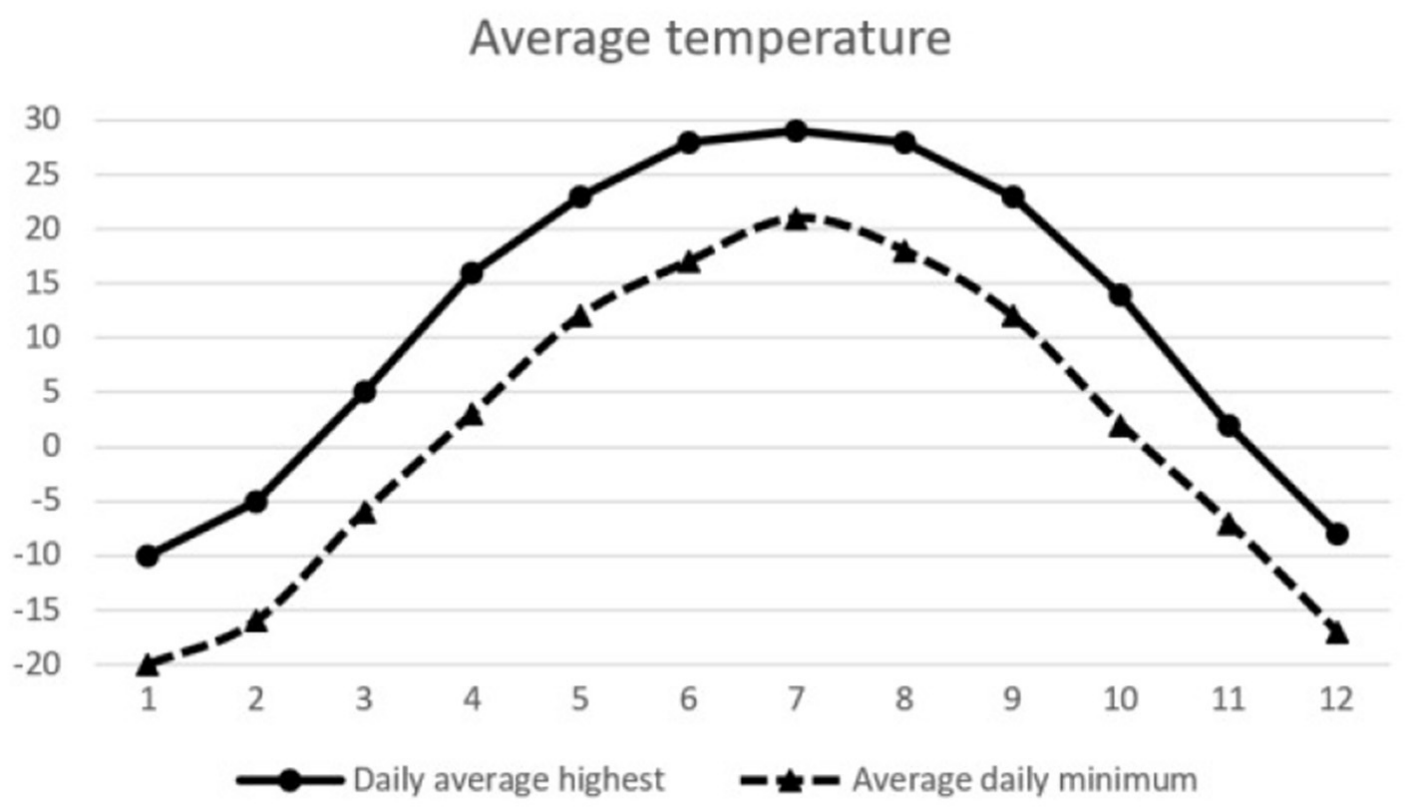
Daily average temperature chart.

For environmental factors, we obtained the temperature and humidity data of Jilin Province from China Meteorological Administration during this period, and the average temperature and humidity of each month in 2019 are shown in Figures 1, 2. For social factors, we collected data on busy farming, suitable school enrollment and festivals. Jilin Province is located in the hinterland of Songliao Plain. It is one of the three famous “golden corn belt” in the world and a traditional granary of China. Jilin Province has a total population of nearly 27 million, of which 11 million are farmers, accounting for 42% of the total population. According to the corn planting cycle, the seasonal labor law data of farmers were generated. The Spring Festival is the most important festival for Chinese people. The total passenger flow during the Spring Festival in 2018 and 2019 totaled nearly 6 billion persons. According to the degree of people's attention to important festivals, the festival importance index data are generated. In addition, the cut-off date for primary school entrance is September 1, that is, two children born in the same year, and who born after September will start school a year later than who born before September. According to the cut-off date of entrance age, periodic data suitable for entrance are generated. Jilin Province is located in the east of mid latitude Eurasia, belonging to the temperate continental monsoon climate, with four distinct seasons, hot and rainy in the same season. It is dry and windy in spring, hot and rainy in summer, clear in autumn and cold in winter. According to the four seasonal variation rules, seasonal periodic data are generated. The data of the above four social factors are shown in Table 1.

**Table 1:** Data of social factors.

**Month**	**1**	**2**	**3**	**4**	**5**	**6**	**7**	**8**	**9**	**10**	**11**	**12**
Farming	0	0	0	0	1	1	1	1	1	1	0	0
Entrance	0	0	0	1	1	1	1	1	1	0	0	0
Season	1	1	2	2	2	3	3	3	4	4	4	1
Festival	1	1	0	0	0	0	0	0	0	0	0	1

### Method

**Figure 3:**
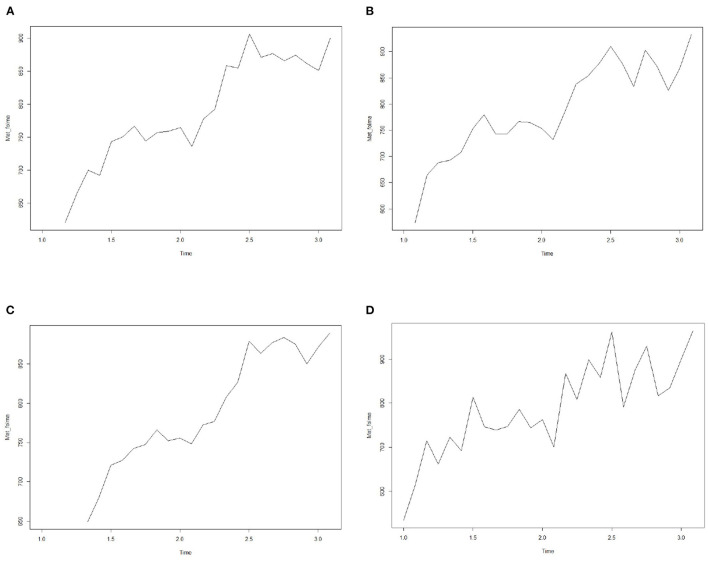
Smooth time series diagram of patient flow. Figures **(A–D)** represent corresponding smoothing curves of order 1, 2, 3 and 5 respectively.

Obstetric patient flow data is a time series data set. Generally, there are obvious errors or random data in the data set. In order to show the trend and seasonal rule of the data set more intuitively, the simplest way is to smooth the time series data set, and then delete these errors and random fluctuations. In this article, SMA function in R software is used to smooth the data set. The four graphs in Figure 3 represent the corresponding smooth curves of order 1, 2, 3 and 5 respectively. It can be seen from the figure that the data has obvious trend and seasonal variation law.

For time series data sets, data can be decomposed into trend component, seasonal component and irregular/error component. Trend component can capture the change of future cycle, seasonal component can capture seasonal change in a cycle, while irregular component can capture those random changes that cannot be explained by trend or seasonal change.

**Figure 4:**
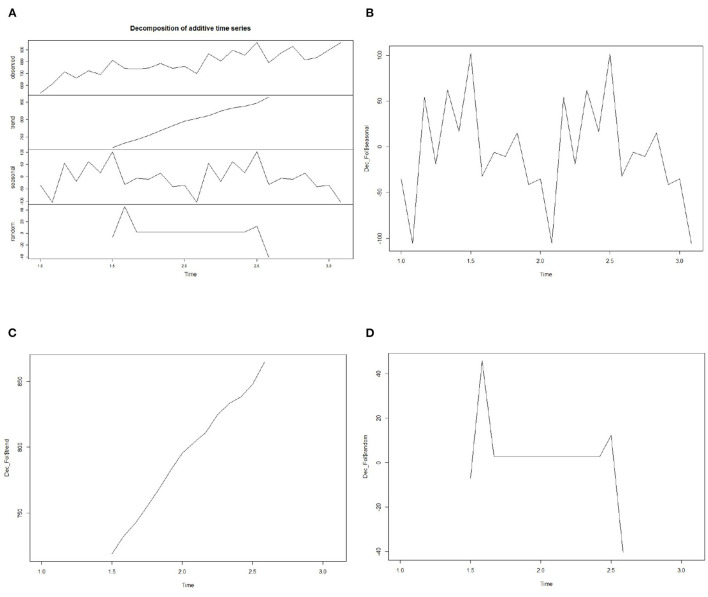
Decomposed patient flow time series diagram. **(A)** decomposition of time series. **(B–D)** seasonal, trend and random section in time series.

In this article, the Decompose function in R software is used to decompose the flow data of patients. The decomposed data are shown in Figure 4A. The x-axis in the figure represents the time period, and the y-axis represents the patient flow. The seasonal section in the figure shows that the flow of pregnant women is peak in autumn and the lowest in winter, as shown in Figure 4B. The flow rate of maternal patients reaches the maximum in July, and shows the minimum value in February. The pregnancy cycle of pregnant women is 40 weeks, totaling 280 days. Deliveries occur in July and February, with the corresponding periods of pregnancy preparation occurring in October and May, that is, the peak of pregnancy preparation in October and the trough in February. In the trend section, the outpatient volume of pregnant women shows a cyclical upward trend over time, as shown in Figure 4C. With the passage of time, the random fluctuation in time series seems to be almost unchanged, presenting a normal distribution rule, as shown in Figure 4D.

### Poisson

The variance of patient flow data was 10,973 and the mean value was 788 by calculating. The variance is much larger than the mean value, which indicates that the model appears excessive dispersion. In this article, the Poisson model of GLM() model is used for analysis. Poisson regression model is most suitable for modeling the events counted by results. More specifically, discrete data with non-negative integer values counts an event, such as the number of events that occur within a given time range or the number of obstetric patients per month.

Poisson regression allows us to determine which explanatory variables affect a given response variable, thus helping us analyze count data. In order to better analyze and predict the flow data of obstetric patients, Poisson regression model is used for univariate and multivariate analyses.

## Results

### Significance Analysis

After univariate and multivariate analyses, there may be significant differences in the results. The inconsistent results can be divided into four cases, which are marked with ABCD in Table 2 for facilitating description during analysis. The results of univariate and multivariate analyses by Poisson regression model are shown in Table 3. After analyzing the significant correlation factors, the Poisson regression model is established. The Predict() function can be used to predict data other than the analysis data.

**Table 2:** Comparison table of significance.

		**Multivariate**	
		**Significance**	**No significance**
Univariate	Significance	A	B
	No Significance	C	D

**Table 3:** Analysis results of significance.

	**Temperature**	**Humidity**	**Farming**	**Entrance**	**Season**	**Festival**
Univariate	1.78E−06	0.000314	7.11E−06	0.112	1.23E−05	0.000167
Multivariate	4.00E−08	0.18403	0.0013	3.51E−10	0.21576	0.00299
Uniformity	A	B	A	C	B	A

The results of univariate and multivariate analyses showed that the significant factors were entrance, busy farming, festivals and temperature. In the analysis results, single factors showed no statistical significance, while multiple factors showed statistical significance, which was marked as B in this article. The possible reason for this situation is that there may be some correlation between this factor and other confounding factors. In univariate analysis, the real effect of this factor was masked by other confounding factors. After eliminating the influence of other factors by multi-factor analysis, it was found that the factor had an independent effect on the dependent variable.

For single factor analysis, there is statistical significance, but multivariate analysis shows no statistical significance, this article marks it as C. the possible reason for this situation is that there may be some false correlation or indirect correlation between independent variables and dependent variables in univariate analysis. For example, factor A has no effect on outcome events, while factor B is an influential factor for outcome events. However, because factor A is only simple and has strong correlation with factor B, there is a collinearity between them. In single factor analysis, there may be significant differences in factor A, leading to factor A being mistakenly considered as an influencing factor and included in multi-factor analysis. In multi-factor analysis, the “false correlation” between factor A and dependent variable disappears by adjusting the influence of factor B. At this time, it can be considered that factor A is not an influencing factor for the dependent variable.

### Validation of Prediction Model

**Figure 5:**
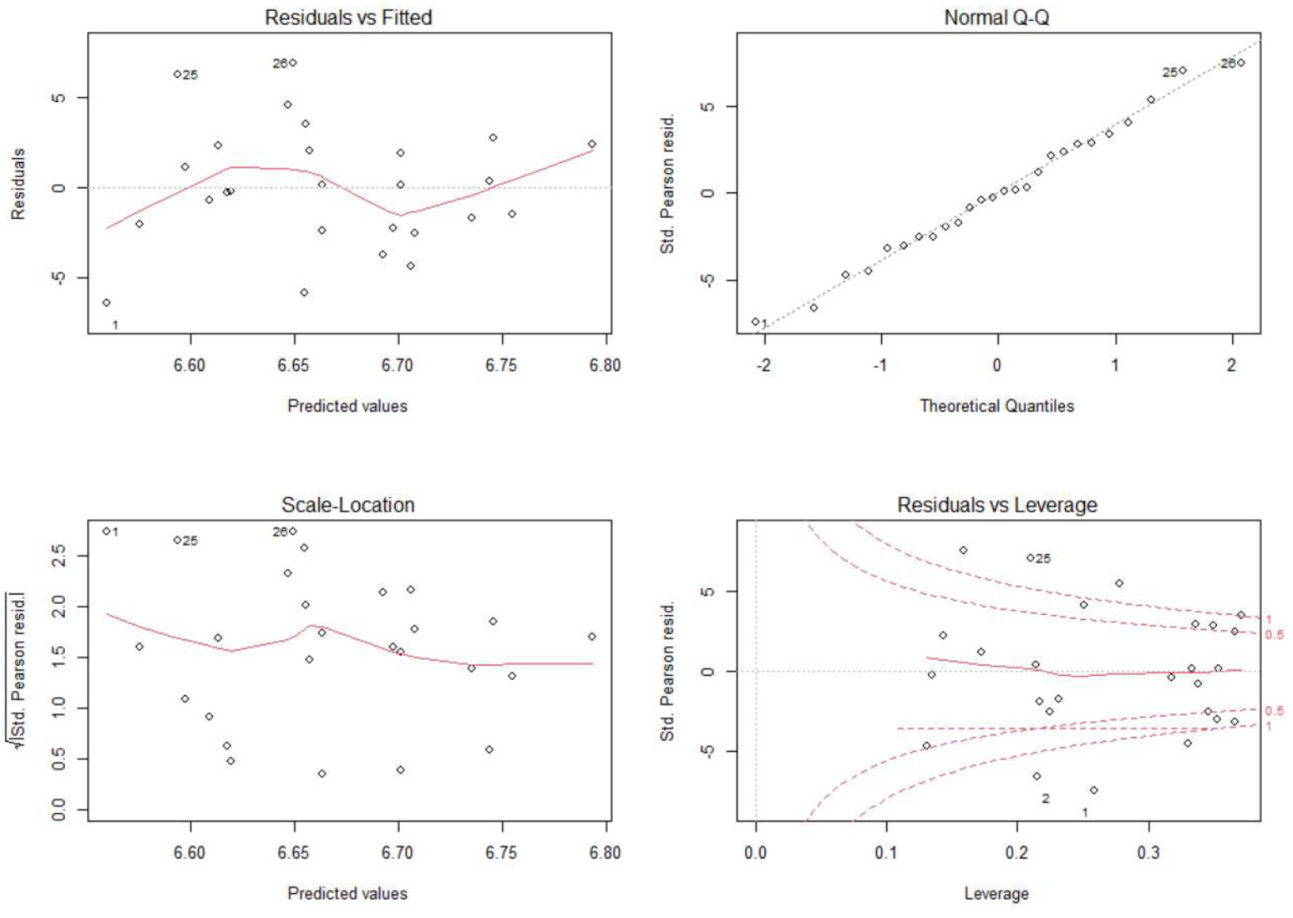
Model verification diagram.

After the establishment of the prediction model, the accuracy of the model is verified by the standards of residual error, constant variance and Cook distance, as shown in Figure 5. In the Residuals vs. fitted graph, the residual is the difference between the predicted values and the real values. This graph shows the relationship between the residual and the real values. It can be seen from the figure that the residual is basically independent of the predicted values. From the Normal QQ diagram, we can see that the residual in the prediction model is normally distributed. From the Scale Location diagram, we can see that the variance is basically a constant, and there is no trend of increasing or decreasing. In this case, we can pass the test of constant variance. In this article, Cook distance is used to analyze whether the data points in the prediction model have significant influence, as shown in the Residuals vs. Leverage diagram. Although some data points are distributed near the contour lines of 0.5 and 1 in the figure, there are not many points too far away from contour line 1. Considering that Cook distance is relative, these data points are not deleted.

In the ideal prediction model, the residual should be a normal distribution, independent of the predicted value. If the residual is still related to the predicted value, it shows that the accuracy of such a model is poor, and there is still room for improvement. From the above experimental results, the prediction model proposed in this article does not need further improvement.

## Discussion

In this article, Poisson regression model is used to obtain the significant factors of maternal patient flow, mainly including entrance, busy farming and festival in social factors and temperature in environmental factors. This part first discusses the domestic and foreign work on the impact of social factors on maternity, and then analyzes and discusses the impact of social factors on maternal. Then, the related work on environmental factors at home and abroad is analyzed. Next, the environmental factors obtained in this article on the impact of temperature on the maternity are discussed. Finally, the limitations of the proposed method are analyzed.

### Social Factors

A large number of studies at home and abroad have shown that, from the perspective of life process epidemiology, the importance of 1,000 days after conception is emphasized and Barker metabolism is supported. The social factors of pregnant women during pregnancy play an important role in influencing the birth and healthy growth of the fetus. Omokanye et al. (20) have analyzed the social factors affecting pregnant women, and found that employment status, birth season, diet, socioeconomic status, income stability, unintended pregnancy, and wealth index are all social factors related to low birth weight. Shore et al.(21) studied the effect of field work on low birth weight in farmers' mothers. Hard field work during pregnancy leads to a high prevalence of low birth weight. Oladeinde et al. (22) analyzed the prevalence and related risk factors of low-birth-weight newborns delivered by traditional midwives, and found that prevalence was significantly associated with maternal age, gestational age, maternal height, marital status, and time of registration. Zhou et al. (23) have studied the impact of short message information on the inappropriate weight of pregnant women in rural China. A set of free short message information mainly includes good family antenatal operation advice and nursing consultation, thus playing a protective role on macrosomia and preventing inappropriate weight during pregnancy. Studies have shown that encouraging care seeking during pregnancy can prevent macrosomia among newborns in rural China. Wang et al. (24) have studied the fertility of female doctors in Taiwan. Their decisions on pregnancy may be affected by long-term medical education and training, leading to the fact that female physicians usually give birth at an older age than non-physicians. However, the age difference between the two groups has gradually declined in the past 20 years. Those who decide health system policies should consider creating a social environment conducive to childbirth for female doctors. Low birth weight is an important factor in neonatal morbidity and mortality. Onalo and Olateju (25) have analyzed the social factors affecting the admission of low-birth-weight infants. In order to reduce the incidence of low birth weight delivery and hospitalization, various interventions have been taken.

After analyzing the data collected on the flow of obstetric patients, this article concludes that the flow of maternal patients reaches its peak in July and the flow of maternal patients reaches its trough in February. Pregnant women have a pregnancy cycle of 40 weeks, a total of 280 days. In July and February, the corresponding pregnancy preparation is in October and May, that is, the peak of pregnancy preparation in October, and the trough in February. The following is to analyze the impact of busy farming on patient flow in social factors. Jilin Province is located in the world-famous “golden corn belt,” with flat terrain and fertile soil. It is an important commodity grain production base in China. The planting time of corn is concentrated in early May, and the harvest time of corn is completed in early October. From planting to harvest, corn has to go through many links, such as weeding, fertilization and pest management. From early May to early October, farmers are working in the field. During pregnancy in May, the field work of the pregnant woman seriously affects the development of the fetus and may even lead to miscarriage. Therefore, the number of pregnant women in May is the least. In early October, when the corn harvest is completed and farmers enter a period of relaxation, pregnancy preparation is conducive to fetal development.

Next, we discuss the impact of social factors, festivals and school entrance on the flow of maternal patients. For Chinese people, the Spring Festival is a year's expectation, and also the strongest time of the year to return home. Even during the Spring Festival, all kinds of traffic is the most congested time, we should try our best to go home for the festival. Every Spring Festival, overpopulated cities such as Beijing, Shanghai, Guangzhou and Shenzhen become almost empty cities. In recent years, the cumulative number of people transported in the Spring Festival has reached nearly 3 billion, which is almost twice the population of China. It is equivalent to that every Chinese has made a round trip between the two places during the Spring Festival. During the Spring Festival, people will go home to reunite with their families, leading to the low flow of pregnant women in February. The following analyses the influence of school admission factors. The cut-off date for primary school entrance is September 1, that is, two children born in the same year, and who born after September will start school a year later than who born before September. As a result, more people are willing to give birth before September.

### Environmental Factors

Global warming is a typical manifestation of observable climate change (26), characterized by the increase of environmental temperature, the increasing frequency and severity of heat waves and extreme hot weather. The WHO estimates that climate change will occur between 2030 and 2050, causing an additional 250,000 deaths each year from malnutrition, malaria, diarrhea and excessive temperatures. The direct cost losses to health by 2030 will be $2 billion to $4 billion a year. The rise of ambient temperature is associated with the development of a variety of acute diseases (including stroke, dehydration, etc.), as well as exacerbating the symptoms of respiratory and psychological diseases, and increasing the risk of death (27). Humidity increase caused by climate change will also pose a threat to human health, including allergic diseases, respiratory diseases (28).

The UK Climate Change Commission's adaptation sub-committee has identified the risk of overheating and poor thermal comfort in hospital buildings (29). Hospitals must provide a comfortable environment for the most vulnerable people in the hottest summer at the time of the year when it is most difficult and the demand for medical treatment is likely to surge. Short-term exposure to high temperatures during heat waves may have adverse effects on birth weight and birth length. Thermal discomfort has a faster effect on performance than cold discomfort. The high temperature environment is associated with the increased risk of work-related injuries, especially in manual labor, where high temperature is a well described occupational hazard (30). Eriksen et al. (31) used multiple regression analysis to study the impact of seasonal factors. The impact of the birth season on the birth weight is greater than on the offspring's own nutrition. The prenatal season environment can change a person's development trajectory and have a long-term impact on health. Sutida et al. (32) analyzed the different temperature ranges acceptable to patients, visitors and medical staff. In order to improve the comfort and health level of residents in medical facilities in tropical areas, it is necessary to carefully integrate and revise the hospital environmental standards according to the different requirements of different medical and health care personnel for thermal comfort. In the hospital environment, the thermal comfort of medical staff is a rarely explored topic. Derks et al. (33) took the nursing staff in the hospital wards as the research object, and analyzed the indoor thermal environment of subjective feedback and measurement. The results show that the design method of dividing hospital wards into different hot areas and setting different levels of comfort for patients and nursing staff seems to be an ideal solution. Qiu et al. (34) used the generalized additive model to control the time trend, meteorological conditions, holidays and days of the week to estimate the correlation. The results indicate that particulate pollution exposure may be an important inducement for hospitalized patients with mental disorders in Chengdu, China, and cause a huge burden of morbidity. Summary articles by Dube et al. (35) notes that more than 30 articles in recent years have discussed the interaction between social and demographic factors and placental diseases. When the concentrations of lead (Pb), arsenic (As) and cadmium (Cd) in the environment exceed the critical limits, these heavy metals are harmful to mothers and infants. Secondly, the most studied social factors include education level, maternal age, prenatal examination rate and parity.

In this article, we get a significant correlation between the flow of maternal patients and the temperature factors in the environment. The following is a detailed discussion based on the year-round temperature situation in Jilin Province. In summer and autumn in Jilin Province, the temperature ranges from 11 to 27 degrees. The temperature ranges from −14 to 2 degrees in spring and winter. After the baby is born in July, the outdoor temperature is appropriate, so you can take the baby to the outdoor sun, which can also effectively avoid the baby's lack of calcium and vitamin D. At this time, the vegetables and fruits that grows naturally are also more conducive to infant vitamin supplement. In other seasons, most babies are faced with large temperature differences between morning and evening, which may lead to respiratory diseases and increase the risk of cold.

### Limitations

The data used in this article is only from a tertiary hospital in Jilin Province, and the data used are less than 3 years, which also affects the significance analysis results of social and environmental factors to a certain extent. This article only analyzes the significant factors of patient flow in obstetrics, and it is also meaningful to analyze the patient flow of other kinds of diseases related to environmental factors. In the future research, it can be extended to more hospitals and more diseases. The analysis of patient flow in multiple hospitals and diseases is of great significance for the government and hospital management to provide decision support. Pregnancy is a long-term process, this article only analyzes the significance of social and environmental factors during childbirth, does not analyze the influencing factors of other stages of pregnancy, and does not analyze the related factors such as air quality.

## Conclusion

In China, improving population quality is an important part of population policy. With the improvement of people's material living standards, people's pursuit of eugenics is constantly improving, especially for couples who have only one child. In this article, Poisson regression model is used to analyze the social and environmental significant factors affecting patient flow, and time series decomposition model is used to analyze the trend and seasonal variation of patient flow, so as to further improve the level of eugenics and pregnancy success rate. The results show that: the environmental and social significant factors affecting the patient flow are busy farming, festival and temperature; the patient flow shows an upward trend every year, accompanied by seasonal changes, with the peak of flow in July and the trough in February. In this article, significant influencing factors, trends and seasonal variation rules of patient flow are analyzed to provide management with decision support, which is conducive to providing pregnant women with higher level of medical services and more comfortable medical experience.

## Data Availability Statement

The datasets presented in this study can be found in online repositories. The names of the repository/repositories and accession number(s) can be found below: https://github.com/hometownjlu/fafm.

## Author Contributions

HL, XY, SW, and DW designed and planned the study. HL, XY, and SW implemented the study and drafted the article. HL and SW analyzed and validated the data under supervision of DM. All authors participated in data analysis and result interpretation, carefully revised all contents of the manuscript, and critically reviewed and approved the submitted manuscript.

## Funding

This work was supported by the National Natural Science Foundation of China (71974074), the Science and Technology Development Plan of Jilin Province (20200301004RQ), and the Education Department of Jilin Province (20211103KJ, 20211106KJ).

## Conflict of Interest

The authors declare that the research was conducted in the absence of any commercial or financial relationships that could be construed as a potential conflict of interest.

## Publisher's Note

All claims expressed in this article are solely those of the authors and do not necessarily represent those of their affiliated organizations, or those of the publisher, the editors and the reviewers. Any product that may be evaluated in this article, or claim that may be made by its manufacturer, is not guaranteed or endorsed by the publisher.
